# Dissecting the Cellular Heterogeneity Underlying Liver Diseases Through the Integration of GWASs and Single-Cell RNA Sequencing

**DOI:** 10.3390/biology14070777

**Published:** 2025-06-27

**Authors:** Miao Zhou, Meng Liu, Chao Xue

**Affiliations:** 1 Zhongshan School of Medicine, Sun Yat-sen University, Guangzhou 510080, China; zhoum223@mail.sysu.edu.cn (M.Z.); lium265@mail2.sysu.edu.cn (M.L.); 2 Medical College, Jiaying University, Meizhou 514031, China; 3 Key Laboratory of Tropical Disease Control (Sun Yat-sen University), Ministry of Education, Guangzhou 510080, China

**Keywords:** liver disease, genome-wide association study, single-cell RNA sequencing, cellular heterogeneity, liver sinusoidal endothelial cells, immune microenvironment

## Abstract

Liver diseases encompass a wide range of etiologies and involve highly heterogeneous cellular environments, yet the specific cellular states through which genetic risk contributes to disease remain incompletely understood. In this study, we integrated genome-wide association study (GWAS) data from six liver diseases and two metabolic traits with transcriptomic profiles of approximately 168,000 human liver cells at single-cell resolution, using the single-cell disease relevance score (scDRS) approach. Our results revealed that disease-associated genetic signals are predominantly localized to non-parenchymal cells—particularly liver sinusoidal endothelial cells (LSECs), cholangiocytes, and specific subsets of lymphocytes. Notably, we identified marked intra-cell-type heterogeneity, with disease associations confined to specific subpopulations exhibiting immune activation or stress-responsive transcriptional programs. For example, autoimmune and viral liver diseases were linked to immunologically active LSECs and cholangiocytes, whereas their metabolically active counterparts showed no enrichment. These findings highlight the necessity of resolving liver cell complexity to uncover the functional basis of genetic risk and suggest that susceptibility to liver disease is driven by specialized cell states within broader cellular categories. Our study provides a refined cellular map of liver disease susceptibility, offering new perspectives for understanding pathogenic mechanisms and informing targeted therapeutic strategies.

## Simple Summary

Liver diseases involve many distinct and functionally diverse cell types, but it remains unclear which specific cellular states contribute to inherited genetic risk. In this study, we integrated genetic data from several liver diseases with single-cell gene expression profiles from human liver tissue to identify disease-associated cells at single-cell resolution. By focusing on cellular heterogeneity, we found that genetic risk is not associated with broad cell types, but with distinct subpopulations defined by their functional states. In particular, liver sinusoidal endothelial cells and cholangiocytes showed disease associations only when in immune-activated or stress-responsive states. For example, autoimmune and viral liver diseases were linked to inflammatory subsets, not their metabolically active counterparts. These findings demonstrate that disease risk is shaped by specific cell states within heterogeneous populations. Our study underscores the need to consider cell-state diversity when studying disease mechanisms and developing targeted therapies.

## 1. Introduction

Liver diseases represent a major global health burden, accounting for over 2 million deaths annually and exhibiting high rates of both morbidity and mortality worldwide [1]. A broad spectrum of liver diseases—including non-alcoholic fatty liver disease (NAFLD), alcoholic liver disease (ALD), viral hepatitis, and autoimmune or cholestatic liver diseases—share complex pathophysiological features and are often influenced by genetic predisposition [2,3,4,5,6]. Genome-wide association studies (GWASs) have identified numerous genetic loci associated with liver disease, providing valuable insights into the underlying genetic architecture [7,8,9,10,11,12]. However, the cellular contexts in which these genetic variants exert their effects remain poorly understood.

The liver is a highly heterogeneous organ comprising diverse cell types—including hepatocytes, liver sinusoidal endothelial cells (LSECs), Kupffer cells (KCs), hepatic stellate cells (HSCs), and cholangiocytes—each contributing uniquely to hepatic homeostasis. This cellular diversity underpins both the physiological complexity of the liver and the heterogeneity characterizing disease mechanisms. Advances in single-cell RNA sequencing (scRNA-seq) have unveiled remarkable transcriptional heterogeneity within the liver, even among traditionally defined cell types, by identifying functionally distinct subpopulations [13,14]. In disease settings, such heterogeneity is closely linked to differential progression patterns and therapeutic responses. For example, central vein-associated HSCs were identified as the principal source of pathogenic collagen production in a murine model of centrilobular fibrosis [15]. Hepatocytes also display significant plasticity, such as transdifferentiation into cholangiocyte-like cells following injury [16], further contributing to disease complexity. This plasticity is critical for liver regeneration and has been implicated in fibrosis progression [17,18,19].

Integrating scRNA-seq with GWASs offers a powerful approach for mapping genetic risk to specific cellular environments. Recent studies have demonstrated that genetic risk variants for hepatocellular carcinoma (HCC) may act through specific immune cell types, such as B cells [20], highlighting the importance of analyzing genetic associations at single-cell resolution. The single-cell Disease Relevance Score (scDRS) framework has emerged as a robust tool to link GWAS signals to specific cell types at single-cell resolution, thereby providing new avenues to elucidate disease mechanisms at the cellular level [21]. This integrative approach helps bridge the gap between genetic associations and functional biology.

In this study, we integrated GWAS summary statistics from six liver diseases and two metabolic traits (mean sample size ≈ 135,000) with scRNA-seq data profiling 168,000 human liver cells. Our goal was to construct a comprehensive, single-cell-resolved map of the cellular contexts in which genetic risk variants for liver disease exert their effects. Specifically, we first identified liver disease-associated cell types and then further dissected intra-cell-type heterogeneity to understand how distinct cellular subpopulations contribute to genetic risk.

## 2. Materials and Methods

### 2.1. Overview of Analysis Workflow

**Figure 1:**
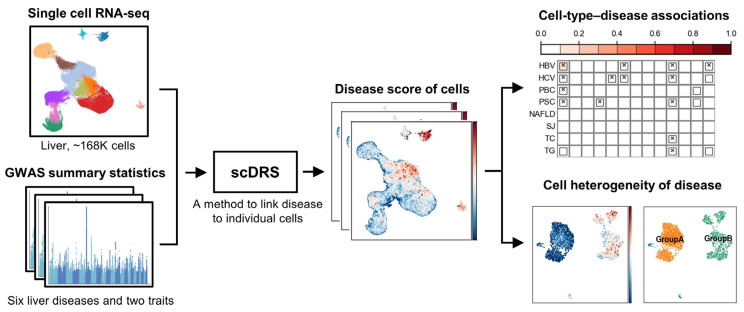
Overview of the analysis workflow.

We integrated single-cell transcriptomic data comprising approximately 168,000 human liver cells with GWAS summary statistics for six liver-related diseases. To associate genetic risk with individual cells, we applied the single-cell Disease Relevance Score (scDRS) method [21], which links disease-associated variants to scRNA-seq profiles at single-cell resolution (Figure 1). For finer analysis, liver cells were grouped into three major classes—non-immune cells, lymphoid cells, and myeloid cells—each analyzed separately for disease. We first assessed associations and heterogeneity at the cell type level and then further investigated heterogeneity within relevant cell types. In addition to six liver diseases, two liver-related metabolic traits were included as reference phenotypes.

### 2.2. Single-Cell RNA Sequencing Data

Single-cell RNA-seq raw count data for ~168,000 cells were obtained from the Liver Cell Atlas (https://www.livercellatlas.org/download.php (accessed on 5 April 2025)) [22]. These cells were derived from 19 individuals and had been pre-clustered and annotated with predefined cell type labels and grouped into three major categories: non-immune (CD45^−^) cells, lymphoid cells, and myeloid cells. We followed the immune cell classification from the original single-cell reference dataset, in which plasmacytoid dendritic cells (pDCs) were classified as part of the lymphoid cells based on transcriptional clustering and developmental similarity, despite not being classical lymphocytes.

### 2.3. GWAS Summary Statistics

GWAS summary statistics for six liver diseases and two metabolic traits were obtained from public repositories (Table 1). Specifically, data for hepatitis B virus infection (HBV) [8], hepatitis C virus infection (HCV) [8], primary biliary cholangitis (PBC) [9], primary sclerosing cholangitis (PSC) [10], and non-alcoholic fatty liver disease (NAFLD) [11] were downloaded from the GWAS Catalog (https://www.ebi.ac.uk/gwas (accessed on 25 March 2025)). Summary statistics for hyaluronan levels in *Schistosoma japonicum* patients (SJ) [23] were obtained from the PMG Lab resource (https://pmglab.top/resource/static/resource/gwas/sj/PMGL_gwas_SJ_HA.sums.tsv.gz (accessed on 26 March 2025)), and data for total cholesterol (TC) and total triglycerides (TG) were downloaded from the UK Biobank Neale Lab (https://www.nealelab.is/uk-biobank (accessed on 27 March 2025)). All GWAS datasets were derived from individuals of European or East Asian ancestry. Linkage disequilibrium (LD) calculations were based on ancestry-matched reference panels from the 1000 Genomes Project Phase 3.

### 2.4. Identification of Disease-Associated Cells Using scDRS

To infer disease-associated cells, we used the scDRS [21], which links scRNA-seq with polygenic disease risk at single-cell resolution. scDRS (version v1.0.2) was obtained from GitHub (https://github.com/martinjzhang/scDRS (accessed on 10 April 2025)). As a prerequisite, gene-level association z-scores were derived using MAGMA (v1.10; https://cncr.nl/research/magma (accessed on 10 April 2025)) [24]. All other scDRS parameters were used with default settings.

### 2.5. Cell Type-Level Association and Heterogeneity Testing

Following cell-level association scoring, we used scDRS to perform statistical tests to assess enrichment of disease signals at the predefined cell type level and to evaluate intra-cell-type heterogeneity. Significance thresholds for both cell-type-level association and heterogeneity were set at a false discovery rate (FDR) < 0.05.

### 2.6. Analysis of Cell-Type Heterogeneity

For cell types showing significant heterogeneity, we performed unsupervised sub-clustering using the Scanpy package (version 1.9.3) [25]. Cells within each heterogeneous cell type were reclustered using the Leiden algorithm on the top 2000 highly variable genes, with the resolution parameter set to 0.5. Based on the distribution of scDRS scores, we merged clusters into transcriptionally and functionally distinct subtypes. To explore subtype-specific functional profiles, the top 200 marker genes of each cluster were subjected to functional enrichment analysis using the g:Profiler web tool (https://biit.cs.ut.ee/gprofiler/gost (accessed on 12 April 2025)) [26]. We selected the Kyoto Encyclopedia of Genes and Genomes (KEGG) [27] and Gene Ontology Biological Process (GO:BP) [28] databases for pathway enrichment.

### 2.7. Code Availability

All scripts used for data processing, statistical analysis, and visualization are publicly available on GitHub at https://github.com/chaoxue-gwas/LiverDiseaseCell (version v1, accessed on 13 April 2025).

## 3. Results

We analyzed scRNA-seq data comprising approximately 168,000 human liver cells obtained from the Liver Cell Atlas (https://www.livercellatlas.org/download.php (accessed on 5 April 2025)) [22]. These cells had been previously clustered and annotated into three major categories: non-immune cells (CD45^−^), lymphoid cells, and myeloid cells. GWAS summary statistics for six liver diseases caused by different etiologies—HBV, HCV, *Schistosoma japonicum* infection (SJ), PBC, PSC, and NAFLD—and two metabolic traits (total cholesterol [TC] and triglycerides [TG]) were collected from publicly available datasets (see Table 1 and Methods for details). We then assessed the associations of these eight phenotypes across the three major cell categories. Our analysis revealed cell-type-specific patterns of genetic association and highlighted substantial cellular heterogeneity at the single-cell resolution.

### 3.1. Association Results in Non-Immune Cells

#### 3.1.1. Association of Non-Immune Cells at the Cell Type Level with Liver Diseases and Traits

**Figure 2:**
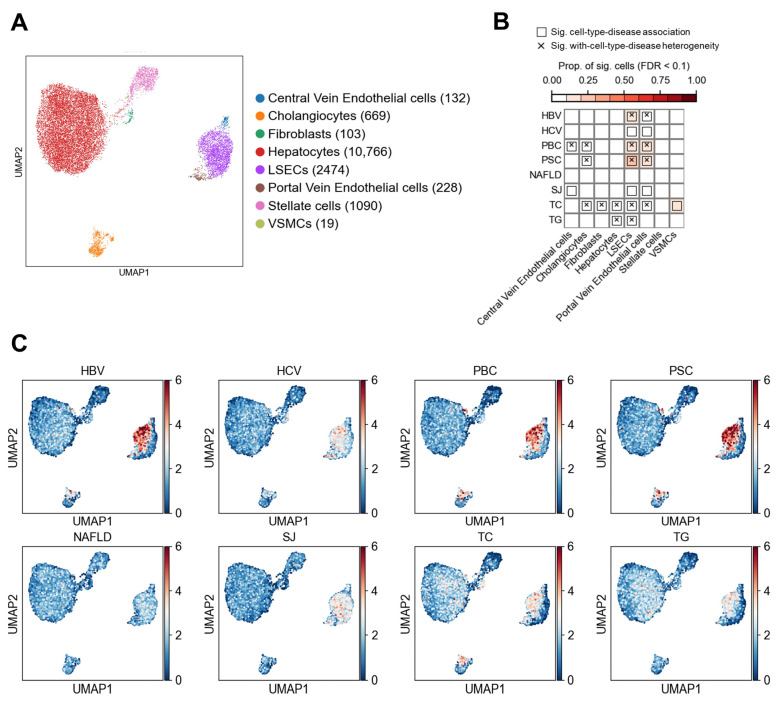
Associations between liver diseases and non-immune cells. (**A**) UMAP visualization of non-immune cells in the liver. Each point represents a single cell, colored by cell type. Numbers in parentheses in the legend indicate the number of cells for each cell type. LSECs, liver sinusoidal endothelial cells. VSMCs, vascular smooth muscle cells. (**B**) Results of association testing and heterogeneity testing between diseases and cell types. The color scale indicates the proportion of cells significantly associated with the disease (FDR < 0.1). Boxes indicate significant associations between diseases and cell types (FDR < 0.05), while crosses indicate significant heterogeneity in disease association among cells within the specified cell type. See the Methods section for explanation of abbreviations. (**C**) Association strength between individual cells and liver diseases or metabolic traits. Each point represents a cell, with coordinates consistent with panel A. Color indicates the association score between the cells and the disease.

A total of 15,481 non-immune cells were analyzed, encompassing eight distinct cell types (Figure 2A). The associations between cell types and diseases/traits are summarized in Figure 2B, while single-cell level associations are shown in Figure 2C. As classical indicators of lipid metabolism, total cholesterol (TC) and triglycerides (TG) are primarily regulated through hepatic synthesis and transport functions. As expected, we observed significant associations of both TC (*P*_TC_ = 0.014) and TG (*P*_TG_ = 0.003) with hepatocytes, supporting the reliability of our integrative analysis. In contrast, none of the six liver diseases showed significant associations with hepatocytes. Instead, HBV, HCV, PBC, PSC, and SJ exhibited strong associations with LSECs and portal vein endothelial cells (PVECs). Additionally, PBC and SJ were significantly associated with central vein endothelial cells, suggesting a critical role of hepatic vascular endothelium in disease pathogenesis. Significant associations were also observed between PBC, PSC, and cholangiocytes, consistent with the cholangiopathic nature of both diseases, which are characterized by bile duct injury and cholestasis. Notably, NAFLD was not significantly associated with any specific non-immune cell type.

**Figure 3:**
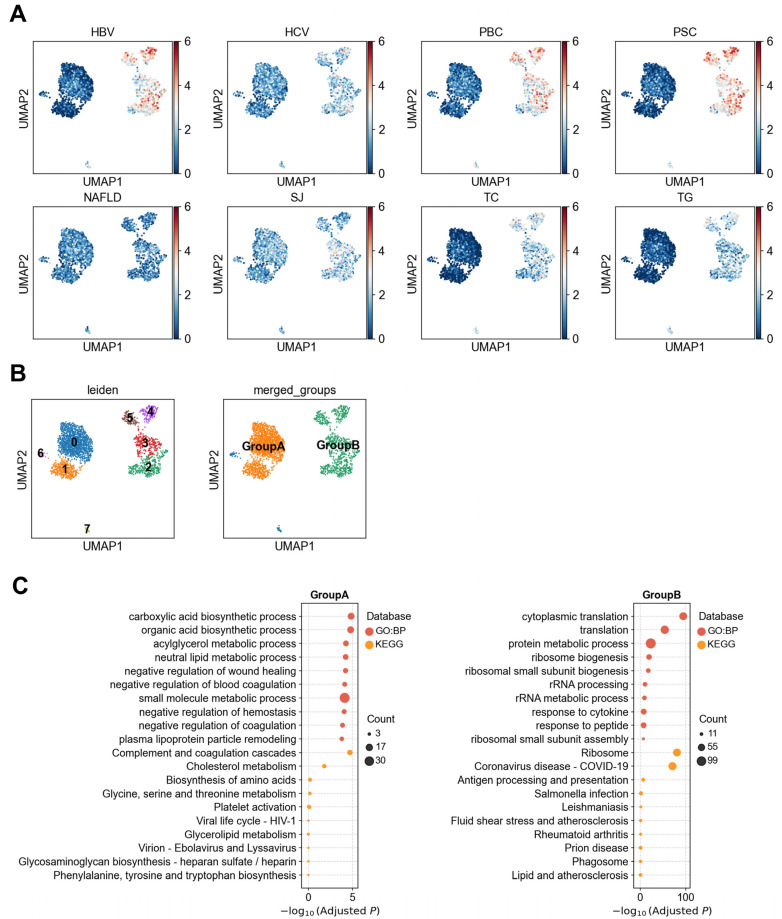
Association analysis of LSECs heterogeneity with liver diseases. (**A**) Association scores between individual liver sinusoidal endothelial cells (LSECs) and liver diseases or metabolic traits. (**B**) Clustering results of LSECs and merged clusters based on similarity in disease association patterns. (**C**) Functional enrichment analysis (GO and KEGG) of differentially upregulated genes between the two LSEC subpopulations, Group A and Group B.

**Figure 4:**
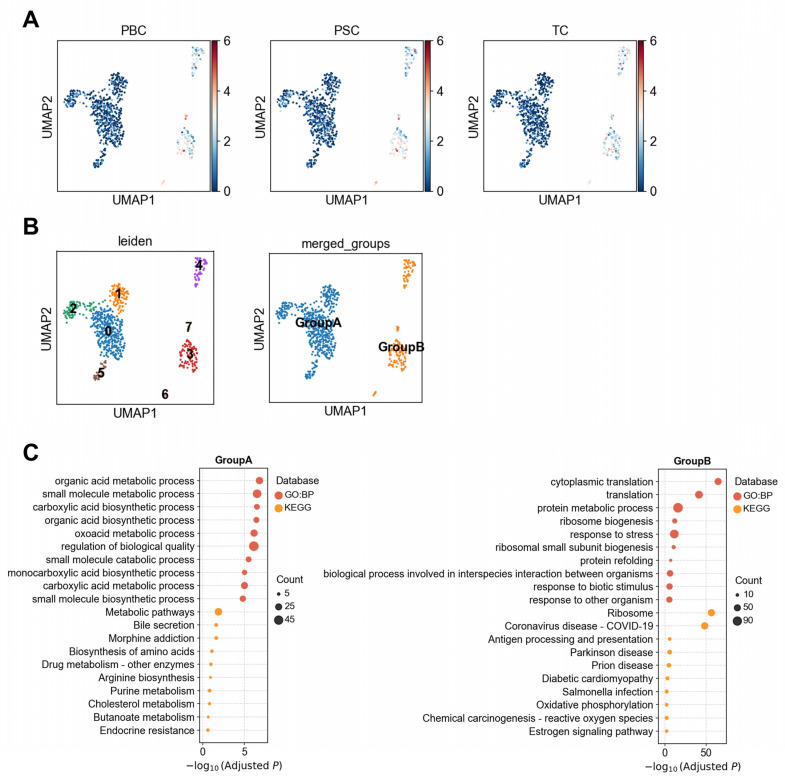
Heterogeneity analysis of cholangiocytes. (**A**) Association scores between individual cholangiocytes and liver diseases or metabolic traits. (**B**) Clustering results of cholangiocytes and merged clusters based on similarity in disease association patterns. (**C**) Functional enrichment analysis (GO and KEGG) of differentially upregulated genes between the two cholangiocyte subpopulations, Group A and Group B.

Among the 14 significant disease–cell type associations identified, 9 exhibited significant cellular heterogeneity (Figure 2B). For example, the associations of LESCs with HBV, PBC, and PSC showed marked heterogeneity across individual cells, with heterogeneity test *p*-values of 0.001 for each. In contrast, although LSECs were significantly associated with HCV and SJ, no evidence of heterogeneity was observed (*P*_heterogeneity_ = 0.16 and 0.25, respectively), indicating homogeneous response across LSEC populations in these cases. Similarly, the associations between cholangiocytes and PBC (*P*_heterogeneity_ = 0.001) as well as PSC (*P*_heterogeneity_ = 0.002) demonstrated marked heterogeneity. To further explore the relationship between cellular heterogeneity and disease risk, we conducted in-depth analyses of LSECs and cholangiocytes as representative cell types (Figure 3 and Figure 4).

#### 3.1.2. Heterogeneity of LSECs in Association with Liver Diseases

To investigate the cellular heterogeneity underlying disease associations within LSECs, we performed reclustering analysis of LSECs. Based on single-cell level disease association patterns (Figure 3A), we observed significant differences in disease association strength across individual cell clusters. Consequently, clusters 0 and 1 were grouped into a subpopulation designated as Group A, while clusters 2–5 were grouped into Group B (Figure 3B). Interestingly, HBV, PBC, and PSC showed stronger associations with Group B compared to Group A, whereas SJ exhibited moderate associations with both subpopulations. To further explore functional differences between these two LSEC subgroups, we performed functional enrichment analysis on the top 200 upregulated differentially expressed genes in these two groups (Figure 3C). Group A cells were significantly enriched in several metabolism-related biological processes, including carboxylic acid biosynthetic process (adjusted *p* = 1.5 × 10^−5^), acylglycerol metabolic process (adjusted *p* = 5.6 × 10^−5^), and cholesterol metabolism (adjusted *p* = 0.015), with the most significantly up-regulated differentially expressed genes in Group A were *GHR* (*p* = 5.3 × 10^−113^) and *APOC3* (*p* = 7.7 × 10^−112^) genes (Supplementary Table S1), suggesting a potential role for this subgroup in lipid synthesis and metabolic regulation.

In contrast, the Group B of LSECs showed significant enrichment in immune- and protein synthesis-related pathways, including cytoplasmic translation (adjusted *p* = 4.9 × 10^−95^), response to cytokine (adjusted *p* = 2.0 × 10^−8^), ribosome (adjusted *p* = 2.6 × 10^−81^), and antigen processing and presentatio*n* (adjusted *p* = 2.4 × 10^−7^) (Figure 3C). These findings suggest that Group B LSECs may play an active role in inflammatory responses and antigen presentation. Notably, *TPT1* was the most significantly upregulated gene in Group B (*p* = 0, Supplementary Table S2). Prior studies report that *TPT1* is known to be highly expressed in cells under oxidative stress, inflammatory stimulation, or undergoing cellular reprogramming [29,30], suggesting that the high-*TPT1*-expressing LSECs subset may represent a functionally remodeled or immunomodulatory cell state. This implies that *TPT1* may serve as a marker or potential target for assessing LSECs state in the context of liver disease.

Collectively, our results indicate that the associations between HBV, HCV, PBC, and PSC and LSECs are confined to a specific subset of immunocompetent LSECs, and are not observed in metabolically active LSEC subsets. In contrast, SJ exhibited moderate associations with both the immunomodulatory and metabolically active LSEC subsets, distinct from the exclusive enrichment pattern of HBV, HCV, PBC, and PSC. This observation implies a more complex or diverse intrahepatic pathogenic mechanism in SJ. These findings highlight the importance of intrinsic functional heterogeneity within LSECs in disease association and suggest that disease-relevant signals may be selectively localized to immunologically active cellular states.

For PVECs, associations were observed with five liver diseases. Among them, three disease-cell type associations showed significant heterogeneity. However, due to the limited number of PVECs, we did not observe any clear enrichment trends within specific cell clusters (Supplementary Figure S1).

#### 3.1.3. Heterogeneous Association Between Cholangiocytes and PBC/PSC Reveals Functionally Distinct Subpopulations

The associations between PBC and PSC with cholangiocytes exhibited significant cellular heterogeneity. To further investigate this, we re-clustered the cholangiocytes (Figure 4A) and grouped the resulting clusters based on their disease association patterns. This yielded two distinct subpopulations: Group A (comprising clusters 0, 1, 2, and 5) and Group B (comprising clusters 3, 4, 6, and 7) (Figure 4B). Interestingly, PBC and PSC had significantly higher correlation with Group B subpopulations than with Group A (Figure 4A). Group A was significantly enriched in pathways related to metabolic homeostasis, including organic acid metabolic process (adjusted *p* = 1.6 × 10^−7^), small molecule catabolic process (adjusted *p* = 3.1 × 10^−7^), metabolic pathways (adjusted *p* = 0.013), and bile secretion (adjusted *p* = 0.024). The most significantly upregulated gene in Group A is *SLC4A4* (*p* = 9.1 × 10^−34^, Supplementary Table S3), which is involved in bicarbonate reabsorption and acid-base balance regulation [31]. Although its expression in cholangiocytes had been rarely documented, its family member *SLC4A2* regulates bile alkalinization through Cl^−^/HCO_3_^−^ exchange in cholangiocytes [32], suggesting that *SLC4A4* may have similar functions in cholangiocytes. These findings indicate that this Group A likely represents metabolically active cholangiocytes involved in bile acid metabolism and detoxification under physiological conditions.

In contrast, Group B exhibited significant enrichment in pathways related to protein synthesis and stress responses, such as cytoplasmic translation (adjusted *p* = 4.3 × 10^−65^) and protein metabolic process (adjusted *p* = 1.8 × 10^−16^) (Figure 4C). Most of the significantly upregulated genes in Group B were ribosomal protein genes (e.g., RPS/RPL families, Supplementary Table S4), which are critical regulators of translation, stress response, cellular proliferation, and repair. These include genes such as *RPL13A* (*p* = 6.38 × 10^−74^), involved in the regulation of inflammatory signaling [33], and *RPS6* (*p* = 6.38 × 10^−74^) and *RPL10* (*p* = 6.38 × 10^−74^), which participate in immune cell differentiation [34]. These data suggest that Group B cholangiocytes may be in a stress-responsive, proliferative, or immune-activated state. Notably, PBC and PSC were specifically associated with Group B, but not Group A, indicating that the disease associations are largely confined to cholangiocytes that adopt an activated phenotype in response to inflammatory cues. This highlights a potential role for Group B cells in the pathogenesis of cholangiopathies and supports the hypothesis that cellular state transitions within cholangiocytes underlie disease-specific susceptibility.

### 3.2. Lymphocyte Subtypes and Their Associations with Liver Diseases

**Figure 5:**
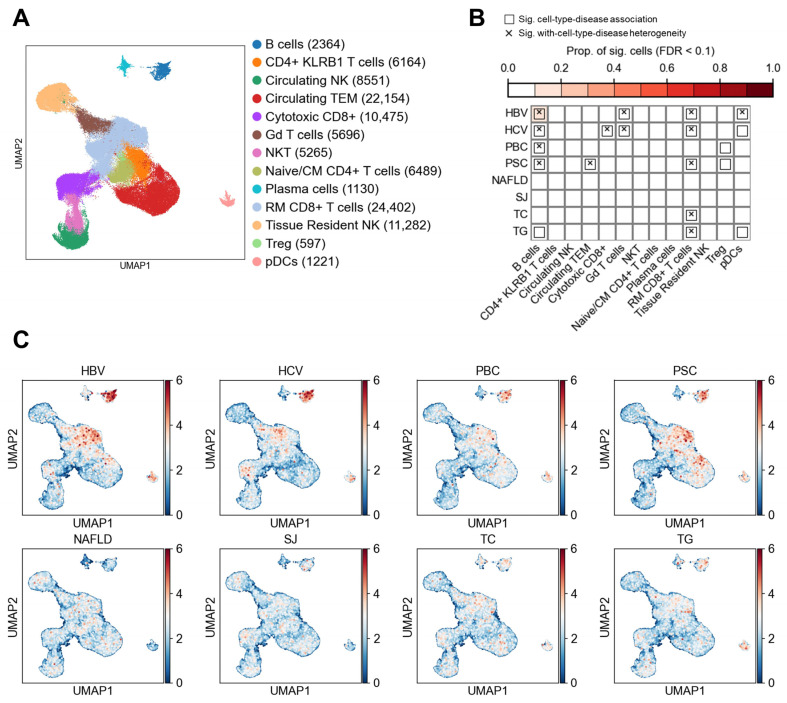
Associations between liver diseases and lymphocytes. (**A**) UMAP visualization of immune cells in the liver. Each point represents a single cell, colored by cell type. Numbers in parentheses in the legend indicate the number of cells for each cell type. (**B**) Results of association testing and heterogeneity testing between diseases and cell types. The color scale indicates the proportion of cells significantly associated with the disease (FDR < 0.1). Boxes indicate significant associations between diseases and cell types (FDR < 0.05), while crosses indicate significant heterogeneity in disease association among cells within the specified cell type. (**C**) Association strength between individual cells and liver diseases or metabolic traits. Each point represents a cell, with coordinates consistent with panel A. Colors indicate the association score between the cell and the disease.

Lymphocytes were subdivided into 13 distinct cell types, including B cells, CD4^+^ KLRB1 T cells, and others (Figure 5A). We identified a total of 15 significant disease–cell type associations (Figure 5B), with single-cell level disease association patterns shown in Figure 5C. Notably, HBV and HCV were significantly associated with Gamma delta (γδ) T cells, resident memory (RM) CD8^+^ T cells, and plasmacytoid dendritic cells (pDCs). These findings are consistent with previous reports highlighting the critical roles of these cell types in antiviral defense and tissue homeostasis [35,36,37,38]. In addition, PBC and PSC showed significant associations with regulatory T cells (Tregs), which are known to be key mediators of immune tolerance and suppressors of excessive inflammation, in line with their established roles in immune regulation during autoimmune liver diseases [39]. Furthermore, significant associations with B cells were observed for HBV, HCV, PBC, and PSC, suggesting a broader involvement of B cell-mediated mechanisms in these disease contexts. No significant associations with lymphocyte subtypes were found for NAFLD or SJ.

**Figure 6:**
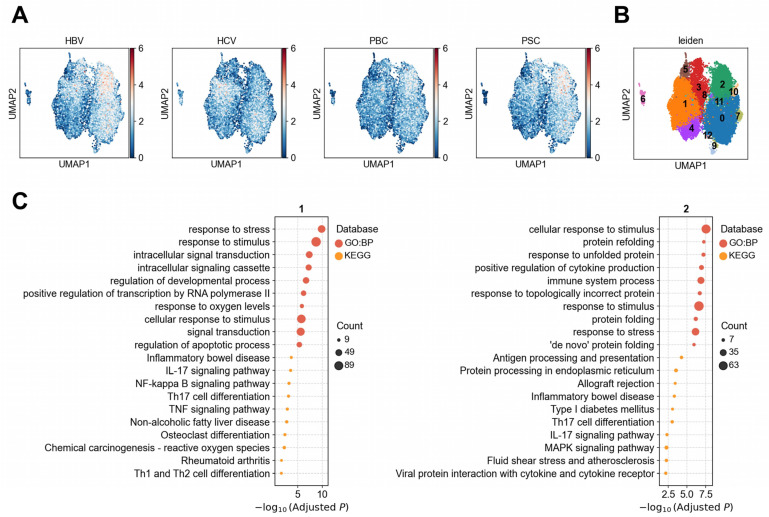
Heterogeneity analysis of RM CD8^+^ T cells. (**A**) Association scores between individual RM CD8^+^ T cells and liver diseases or metabolic traits. (**B**) Clustering results of RM CD8^+^ T cells. (**C**) Functional enrichment analysis (GO and KEGG) of differentially upregulated genes between the two cell clusters, Cluster 1 and Cluster 2.

Among the 15 observed disease–cell type associations, 12 showed evidence of cellular heterogeneity (Figure 5B). Notably, associations between HBV, HCV, and PSC with RM CD8^+^ T cells exhibited significant heterogeneity. We performed reclustering of RM CD8^+^ T cells and, based on the disease–cell association patterns at the single-cell level (Figure 6A), identified two major cell clusters—Cluster 1 and Cluster 2—that accounted for the majority of cells expressing disease-associated genes. HBV and PSC showed stronger associations with Cluster 2, while HCV was predominantly associated with Cluster 1. To explore the functional characteristics of these two subpopulations, we conducted differential expression and enrichment analyses for each cluster (Figure 6B). Cluster 1 was significantly enriched in pathways related to immune response, inflammation, and signal transduction, such as the IL-17 signaling pathway (adjusted *p* = 2.7 × 10^−4^), NF-kappa B signaling pathway (adjusted *p* = 5.9 × 10^−4^), and Th17 cell differentiation (adjusted *p* = 7.3 × 10^−4^). The most significantly upregulated genes in Cluster 1 included mitochondrial-encoded genes (e.g., *MT-ATP6*, *MT-CO3*, *MT-CO2*, *MT-ND2*, *p* = 0, Supplementary Table S5) and the oxidative stress regulator *TXNIP* (*p* = 0, Supplementary Table S5). These genes are commonly co-expressed in lymphocytes under inflammatory, viral, or chronic stress conditions, indicating a high metabolic and oxidative stress regulatory state [40]. In addition, *IL7R* and *FOSB* were also upregulated, suggesting an immune-active or activation-prone phenotype in this subpopulation. In contrast, Cluster 2 was enriched in biological processes related to protein folding, antigen processing, and immune regulation, including protein refolding (adjusted *p* = 5.9 × 10^−8^) and antigen processing and presentation (adjusted *p* = 5.4 × 10^−5^). The most highly upregulated genes in this cluster comprised chaperone/heat shock proteins (e.g., *HSPA1A/HSPA1B*, *DNAJB1*, *HSPE1*, *p* = 0), immune effectors (e.g., *IFNG*, *KLRB1*, *p* = 0), and ribosomal/protein synthesis factors (e.g., *RPL31*, *p* = 0) (Supplementary Table S6). These genes likely support the survival, rapid responsiveness, and sustained effector function of RM CD8^+^ T cells under hypoxic or stress conditions. Although other disease–lymphocyte associations, such as those involving B cells in HBV, HCV, PBC, and PSC, and γδ T cells in HBV and HCV, also displayed signs of cellular heterogeneity, we did not observe clear enrichment within specific subpopulations (Supplementary Figure S1).

### 3.3. Myeloid Cell Associations

**Figure 7:**
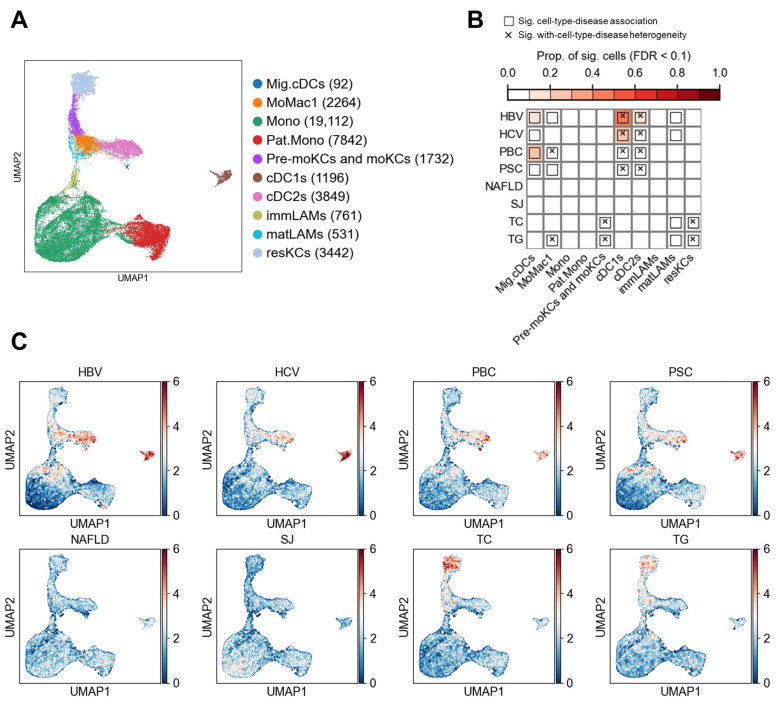
Associations between myeloid cells and liver diseases or traits. (**A**) UMAP visualization of myeloid cells in the liver. Each point represents a single cell, colored by cell type. Numbers in parentheses in the legend indicate the number of cells for each cell type. (**B**) Results of association testing and heterogeneity testing between diseases and cell types. The color scale indicates the proportion of cells significantly associated with the disease (FDR < 0.1). Boxes indicate significant associations between diseases and cell types (FDR < 0.05), while crosses indicate significant heterogeneity in disease association among cells within the specified cell type. (**C**) Association strength between individual cells and liver diseases or metabolic traits. Each point represents a cell, with coordinates consistent with panel A. Colors indicate the association score between the cell and the disease.

Myeloid cells were categorized into 10 distinct cell types (Figure 7A), including migratory dendritic cells (Mig.cDCs) and monocyte-derived macrophage cluster 1 (MoMac1). The associations between these cell types and liver diseases are summarized in Figure 7B, revealing 17 significant disease–cell type associations. Single-cell level associations with diseases are shown in Figure 7C. Notably, HBV, HCV, PBC, and PSC were significantly associated with multiple dendritic cell subsets, including Mig.cDCs, conventional dendritic cells type 1 (cDC1s), and cDC2s (Figure 7B). These findings suggest that dendritic cells may contribute to disease pathogenesis and progression through their potent antigen-presenting capacity, immune activation, and regulatory functions, consistent with previous studies [41]. No significant associations were observed between NAFLD and SJ and any myeloid cell types. In contrast, TC and TG were significantly associated with three subsets of Kupffer cells (pre-monocyte-derived and monocyte-derived Kupffer cells [Pre-moKCs and moKCs], mature lipid-associated macrophages [matLAMs], and resident Kupffer cells [resKCs]), indicating a potential link between myeloid immune cells and lipid metabolism. Although cellular heterogeneity was observed within cDC1s and cDC2s—both of which were associated with HBV, HCV, PBC, and PSC—no specific disease-enriched subpopulations were identified (Supplementary Figure S1).

## 4. Discussion

Liver diseases are a major global health burden, and understanding the cellular context of genetic susceptibility is essential to elucidate their pathogenesis and improve targeted interventions [1]. In this study, we systematically integrated GWAS data from six liver diseases and two metabolic traits with scRNA-seq data from approximately 168,000 human liver cells. This approach enabled the construction of a cell-type-specific risk map at single-cell resolution and facilitated the exploration of functional heterogeneity within key cellular subpopulations. Our findings not only expand the current understanding of the genetic basis of liver diseases but also reveal how disease-associated genetic risks manifest in a cell-type-specific and heterogeneous manner, offering new insights into the mechanisms of liver disease pathogenesis.

We first observed that metabolic traits—specifically TC and TG—were strongly associated with hepatocytes, consistent with their central role in lipid metabolism [42]. This result serves as a positive control, validating the robustness of our analytical framework. Interestingly, contrary to conventional expectations, most liver diseases exhibited significant associations not with hepatocytes or Kupffer cells (traditionally considered key targets), but rather with non-parenchymal cell types, including LSECs, portal endothelial cells, cholangiocytes, and immune cells. These findings highlight the critical roles of non-parenchymal compartments in the onset and progression of liver diseases and align with recent insights emphasizing the importance of the hepatic microenvironment and intercellular interactions in disease mechanisms [43,44]. In contrast, we found no significant association between NAFLD and any specific liver cell type. This may reflect the multifactorial and multicellular nature of NAFLD, which is often driven by systemic metabolic disturbances and complex intercellular crosstalk. Alternatively, the lack of signal may be due to the limited statistical power or the polygenic architecture of the current GWAS dataset for NAFLD.

Notably, our findings revealed that diseases such as HBV infection, PBC, and PSC were predominantly associated with a specific subpopulation of LSECs, designated as Group B, which is enriched for immune-related gene expression. In contrast, another LSEC subpopulation (Group A), characterized by genes involved in lipid metabolism, showed no significant disease association. Group B exhibited high expression of *TPT1*, ribosomal genes, and genes related to antigen presentation, suggesting a critical role in antigen processing, immune activation, and stress regulation [45,46]. The unique fenestrated structure of LSECs allows direct exposure of the liver to gut-derived microbial and dietary antigens via the portal vein [47]. Although traditionally regarded as structural mediators and facilitators of lipid transport, LSECs have increasingly been recognized for their active role in antigen presentation and immune tolerance [48,49]. Our findings provide further support for this emerging paradigm and, importantly, offer the first genetic evidence for functional heterogeneity within LSEC subpopulations in the context of liver disease risk.

Similarly, in both PBC and PSC, disease associations were specifically enriched in a subpopulation of cholangiocytes (Group B) characterized by high expression of ribosomal protein genes. These cells exhibited transcriptional signatures indicative of cellular stress, metabolic reprogramming, and elevated protein synthesis activity. This aligns with previous reports highlighting the role of cholangiocyte stress responses and regenerative programs in the context of cholestasis and immune-mediated injury [50,51]. Our findings provide novel insights into the cellular basis of cholangiopathies, underscoring the relevance of stress-responsive cholangiocyte subpopulations in the pathogenesis of biliary diseases.

Within lymphocyte populations, we observed significant enrichment of genetic risk for HBV, HCV, PBC, and PSC in specific subpopulations of RM CD8^+^ T cells. These enriched subclusters exhibited functional signatures associated with immune activation, heightened mitochondrial activity, and enhanced antigen presentation, which are consistent with their established roles in chronic inflammation and viral control [52]. Notably, in PSC, the RM CD8^+^ T cell subpopulation enriched for disease risk also expressed elevated levels of heat shock proteins and cytotoxic molecules such as *IFNG* and *KLRB1*, suggesting a state of persistent activation. This persistent immune activity may exert chronic immunological pressure on the biliary epithelium, contributing to the ongoing tissue injury and inflammation characteristic of PSC [53].

In the myeloid compartment, multiple liver diseases showed genetic risk enrichment in dendritic cell subsets, including cDC1s, cDC2s, and MigDCs, suggesting a shared involvement of antigen presentation and adaptive immune activation mechanisms across different hepatic pathologies. However, we did not observe stable enrichment patterns within specific dendritic cell subclusters. This may reflect the continuum of transcriptional states and the dynamic functional transitions characteristic of these cells [54].

Notably, significant genetic risk enrichment for NAFLD and schistosome infection was not observed in most cell types, implying that these conditions may be more strongly influenced by environmental factors or that their genetic effects are mediated through alternative mechanisms such as epigenetic regulation. Further investigation is warranted to elucidate these possibilities.

Overall, our study highlights the cell type specificity and subpopulation-level functional heterogeneity of genetic risk for liver diseases at single-cell resolution. These findings demonstrate that disease-associated genetic variants preferentially exert their effects within distinct cellular subpopulations defined by specific functional states, such as activation, stress response, or immune engagement. This suggests that genetic risk is not uniformly distributed across entire cell types but is instead concentrated in dynamic and functionally specialized subsets. This observation aligns with evidence from single-cell eQTL analyses indicating that genetic variants influence gene expression in a cell state-dependent manner [55], offering a more nuanced understanding for pinpointing therapeutic targets.

This study has several limitations that warrant cautious interpretation. First, the scDRS framework links disease risk with cells based on the similarity between disease-associated genetic signals and cell-specific gene expression profiles. As such, it captures correlation rather than causation, and the identified associations do not imply direct functional involvement of these cell types in disease etiology. Second, our analysis relies solely on transcriptomic data, which may not fully reflect the molecular complexity of disease-relevant cells. Other regulatory layers, such as protein abundance, chromatin accessibility, and epigenetic modifications, likely contribute to disease mechanisms and are not captured in this study. Third, conventional single-cell RNA-seq lacks spatial resolution, limiting our ability to assess how microenvironmental context or tissue architecture influences cellular heterogeneity and disease risk. Future studies integrating spatial transcriptomics, proteomics, and epigenomic profiling at single-cell resolution will be essential to delineate causal pathways and identify context-specific therapeutic targets with greater precision.

In summary, this study systematically mapped the genetic risk of liver diseases across the cellular landscape at single-cell resolution. Our results underscore the importance of intra-cell-type heterogeneity in mediating disease susceptibility. This cell-level genetic risk atlas not only enhances our understanding of liver disease pathogenesis but also provides a valuable reference for future studies aiming to identify functional target cells and design precision therapeutic strategies.

## 5. Conclusions

By integrating GWAS data from multiple liver diseases and metabolic traits with human liver scRNA-seq data, we constructed a high-resolution cell-type-specific disease atlas. Our results demonstrate that genetic risks for liver diseases are not uniformly distributed across canonical cell types but are often restricted to specific functional subpopulations, particularly those involved in immune activation, stress response, or regeneration. These findings highlight the utility of single-cell transcriptomic approaches in functional genomics and provide important cellular contexts for future mechanistic studies and therapeutic exploration in liver diseases.

## Figures and Tables

**Table 1:** Summary of GWAS data for liver diseases and traits.

Disease/Trait Name	Abbreviation	Population	Sample Size	PMID
Hepatitis B virus infection	HBV	East Asian	171,822	34594039
Hepatitis C virus infection	HCV	East Asian	176,698	34594039
Primary biliary cholangitis	PBC	European	24,510	34033851
Primary sclerosing cholangitis	PSC	European	14,890	27992413
Non-alcoholic fatty liver disease	NAFLD	European	9677	31311600
*Schistosoma japonicum* hyaluronan levels	SJ	East Asian	637	35493725
Cholesterol	TC	European	340,162	25826379
Triglycerides	TG	European	340,162	25826379

PMID indicates the PubMed ID of the publication from which the GWAS data were obtained.

## Data Availability

The original contributions presented in this study are included in the article/Supplementary Materials. Further inquiries can be directed to the corresponding authors.

## Supplementary Materials

The following supporting information can be downloaded at: https://www.mdpi.com/article/10.3390/biology14070777/s1, Figure S1: Associations between individual cells and disease within heterogeneous cell types; Table S1: Top 200 upregulated genes in the Group A cell subpopulation of LESCs; Table S2: Top 200 upregulated genes in the Group B cell subpopulation of LESCs; Table S3: Top 200 upregulated genes in the Group A cell subpopulation of cholangiocytes; Table S4: Top 200 upregulated genes in the Group B cell subpopulation of cholangiocytes; Table S5: Top 200 upregulated genes in the Cluster 1 cell subpopulation of RM CD8^+^ T cells; Table S6: Top 200 upregulated genes in the Cluster 2 cell subpopulation of RM CD8^+^ T cells.

## Abbreviations

The following abbreviations are used in this manuscript:

GWAS	Genome-wide association study
scRNA-seq	Single-cell RNA-sequencing
LD	Linkage disequilibrium
GO	Gene Ontology
HBV	Hepatitis B virus infection
HCV	Hepatitis C virus infection
PBC	Primary biliary cholangitis
PSC	Primary sclerosing cholangitis
NAFLD	Non-alcoholic fatty liver disease
SJ	Schistosomiasis progression
TC	Cholesterol
