# Supplementary material for: Dissecting the Cellular Heterogeneity Underlying Liver Diseases Through the Integration of GWASs and Single-Cell RNA Sequencing
Source: Biology (Basel). 2025 Jun 27;14(7):777. doi: 10.3390/biology14070777 (PMC12293027; doi:10.3390/biology14070777)
Supplement: Supplementary file 1 [file biology-14-00777-s001.zip › biology-3709905-supplementary/Supp/SuppFigureS1.pdf]

Disease score of Pre-moKCs and moKCs

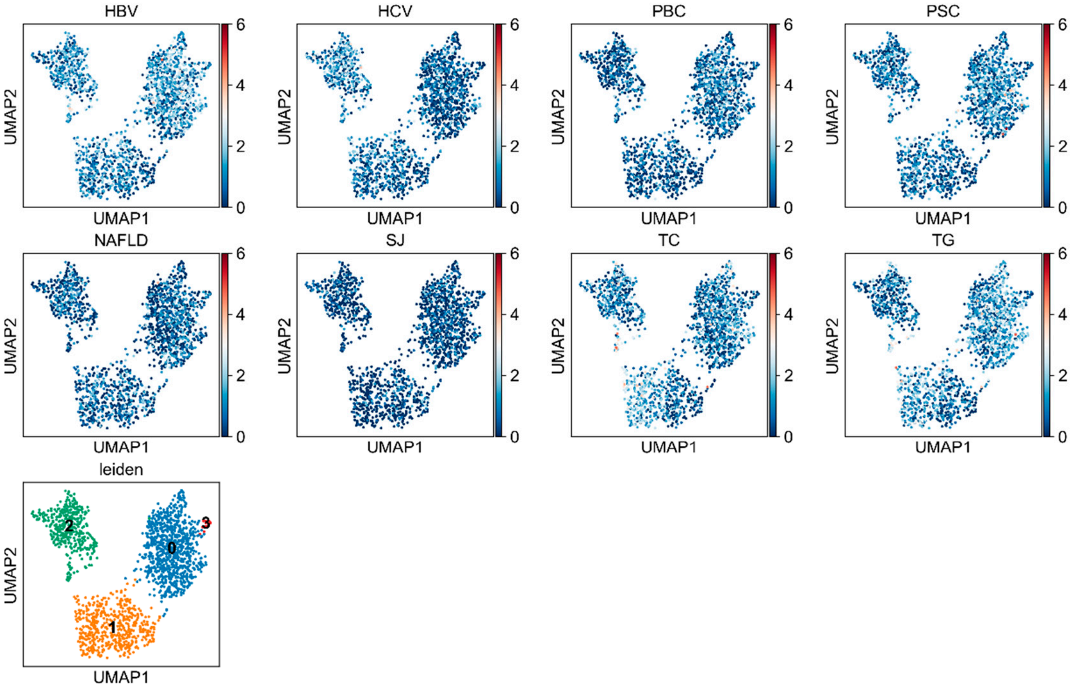

Disease score of MoMac1

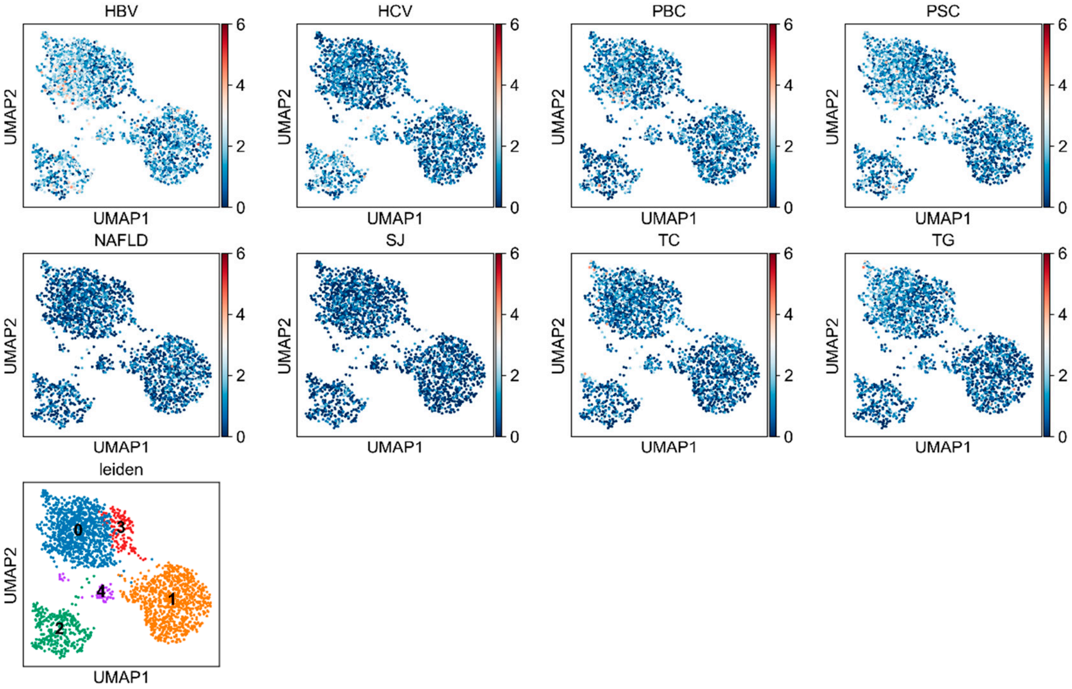

Disease score of cDC2s

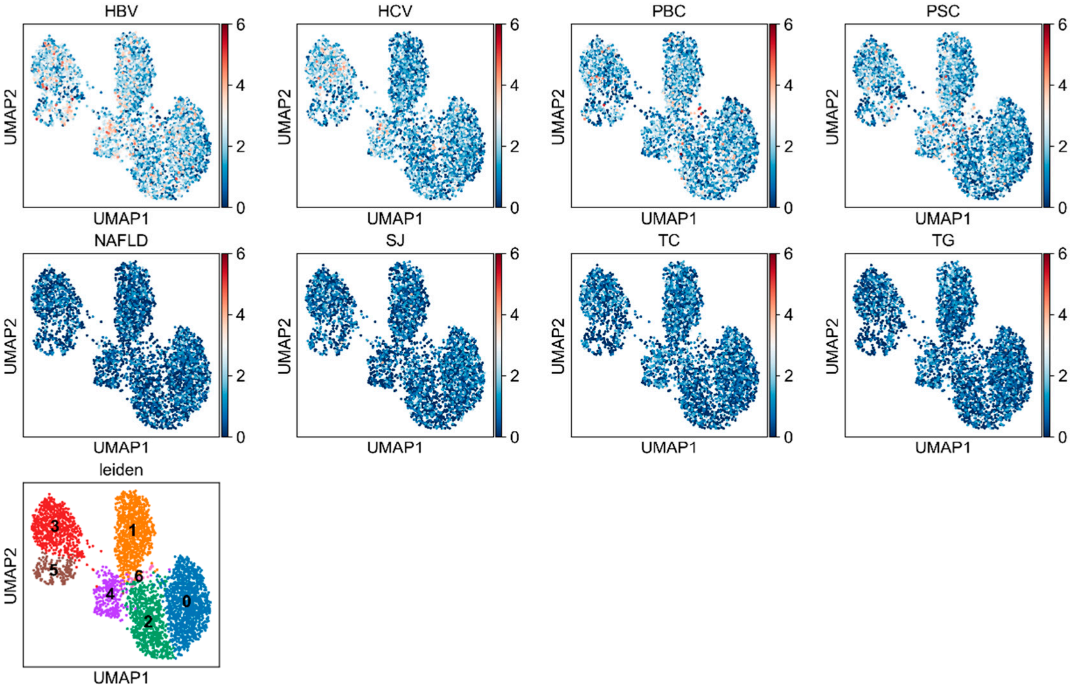

Disease score of cDC1s

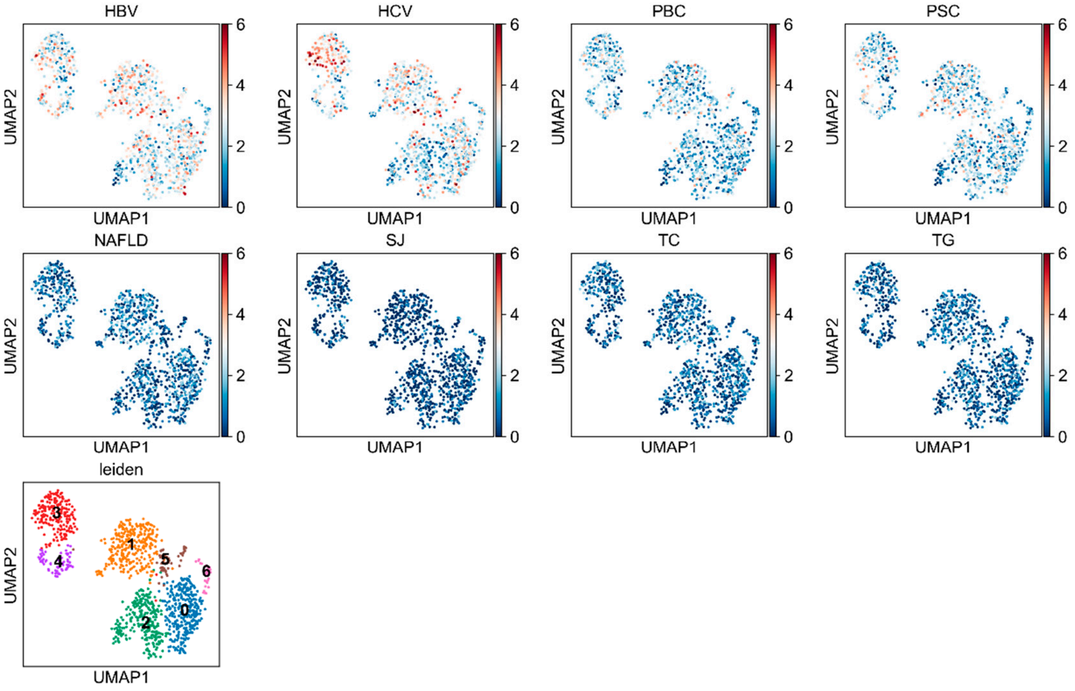

Disease score of RM CD8+ T cells

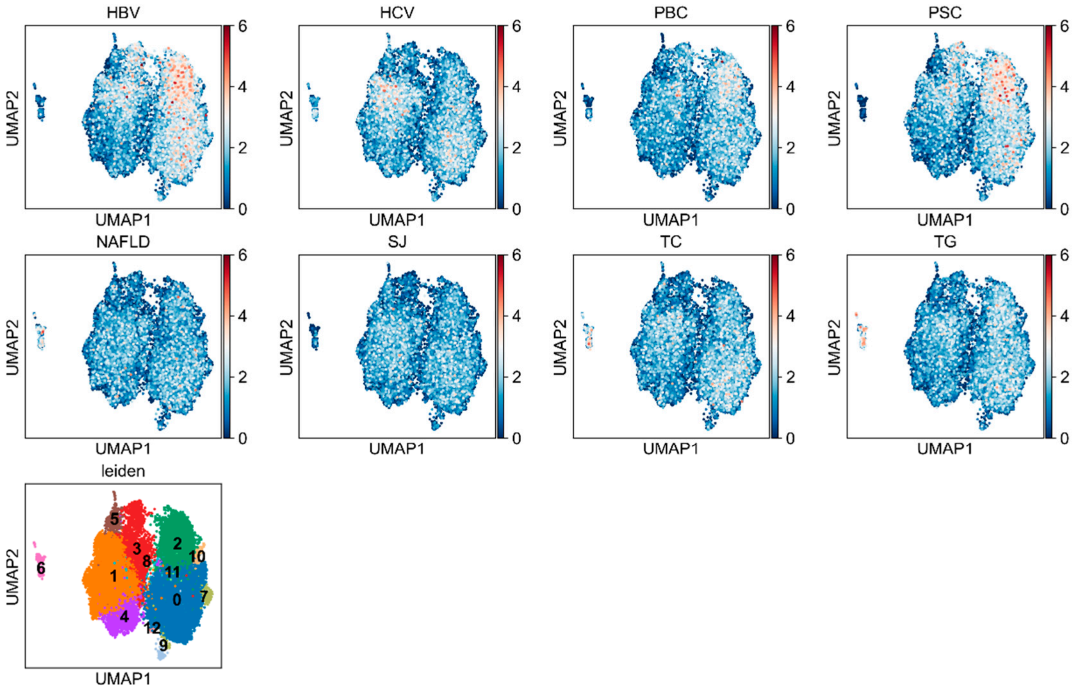

Disease score of pDCs

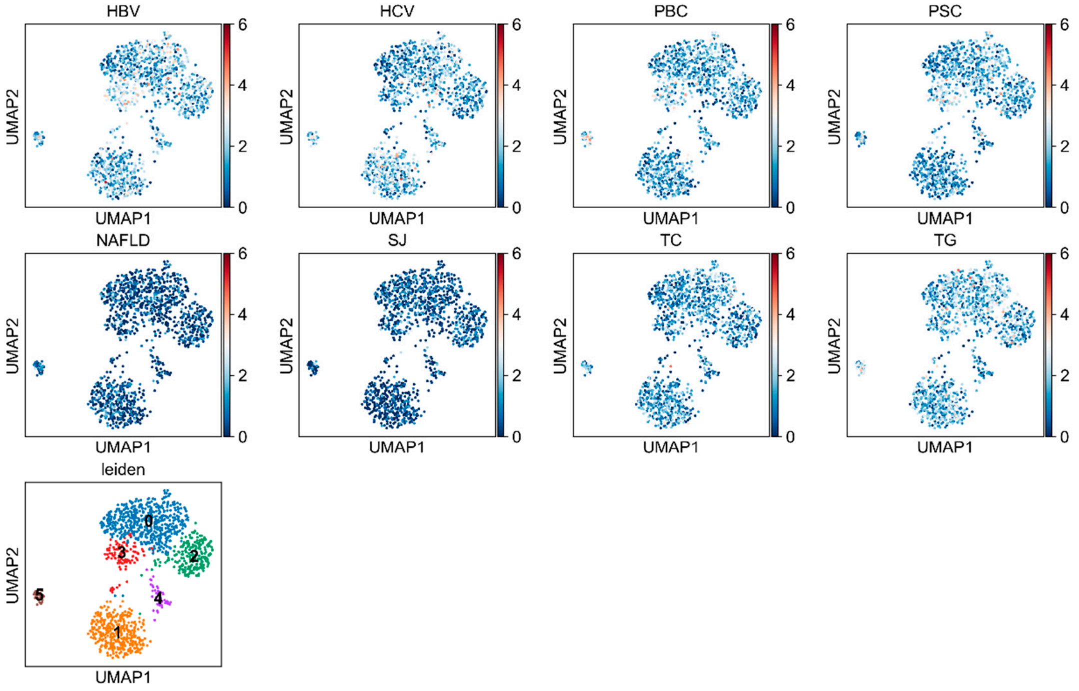

Disease score of Gd T cells

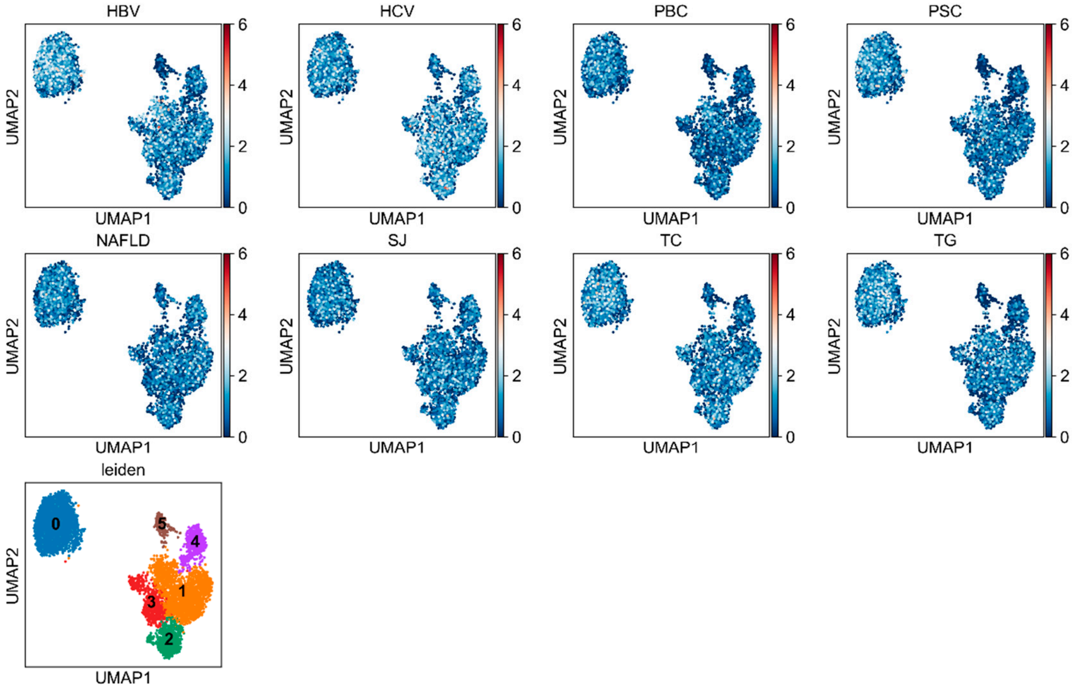

Disease score of Cytotoxic CD8+

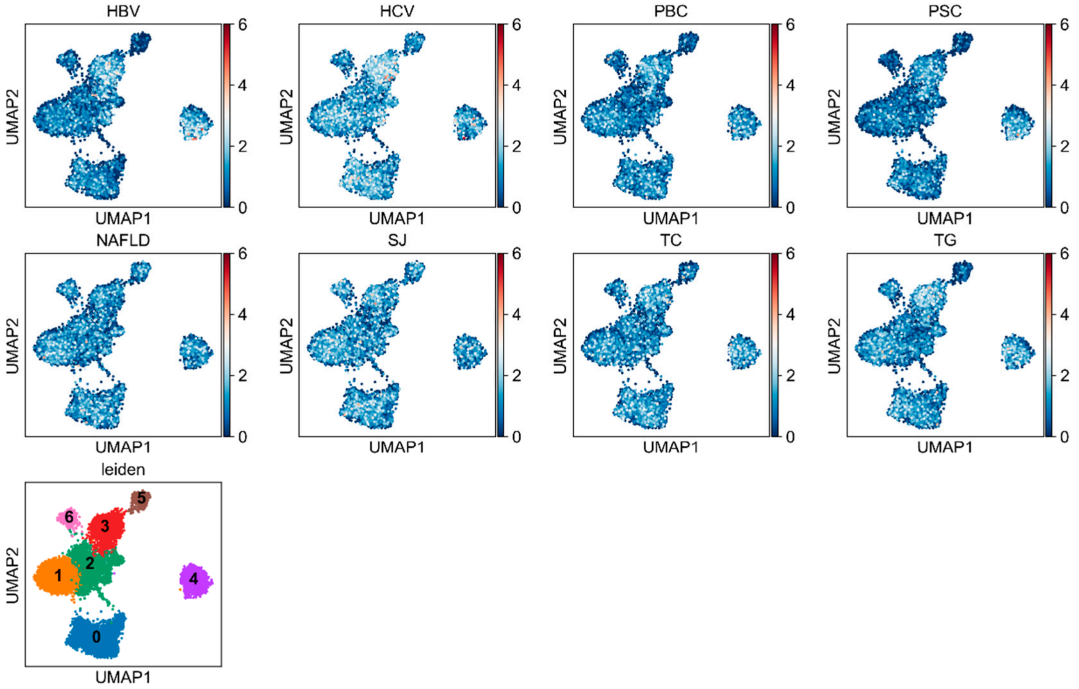

Disease score of Circulating TEM

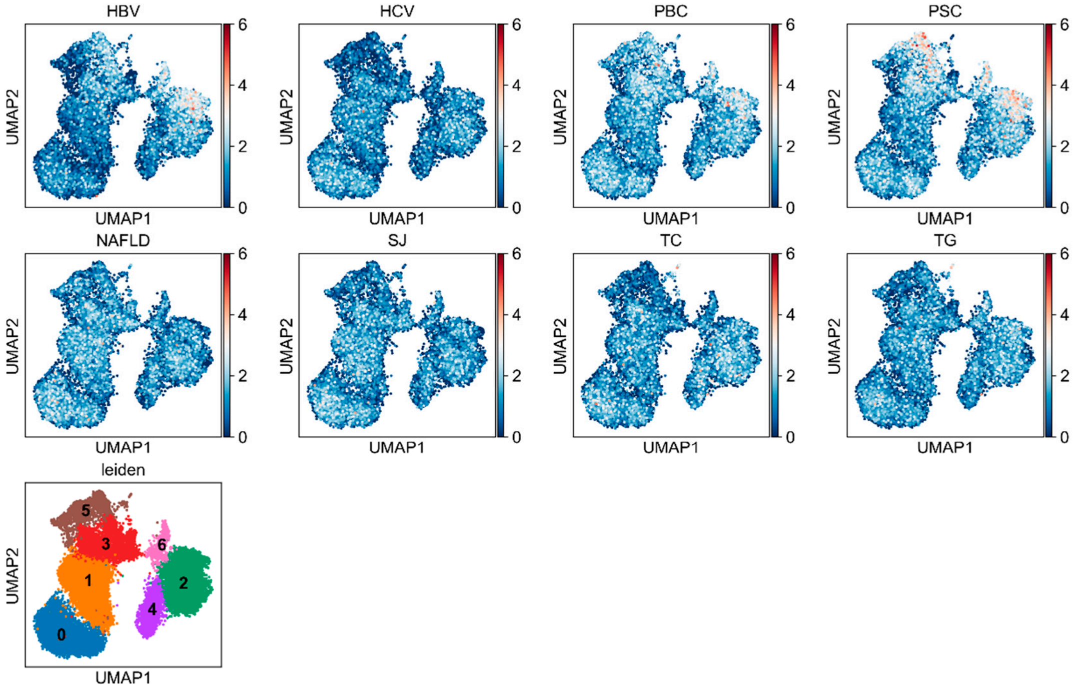

Disease score of B cells

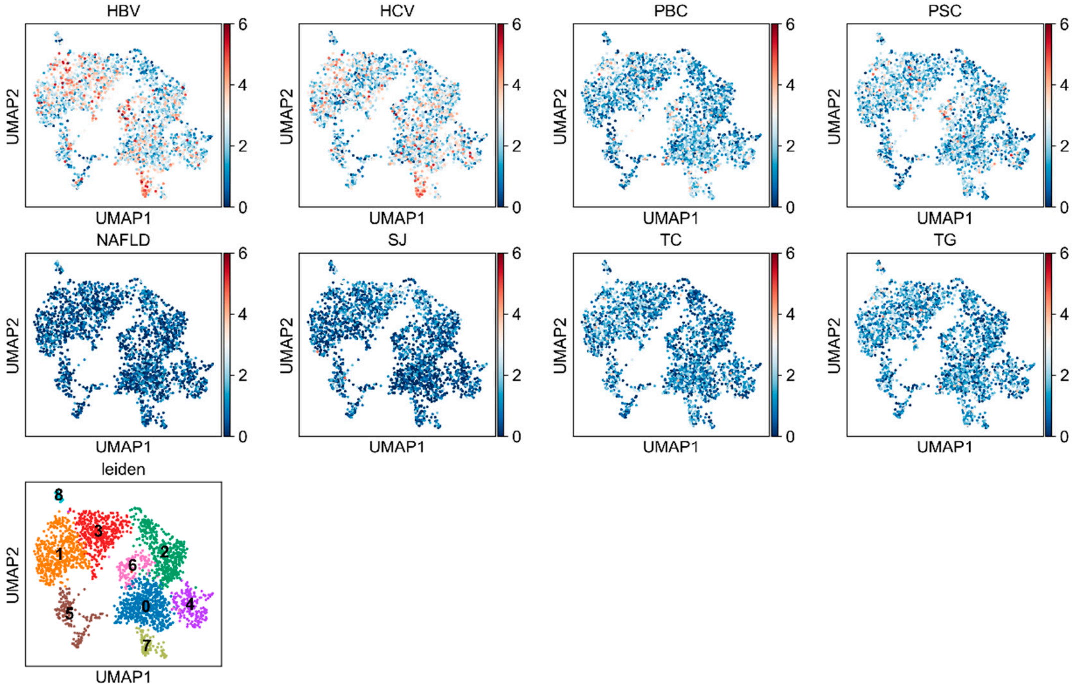

Disease score of Portal Vein Endothelial cells

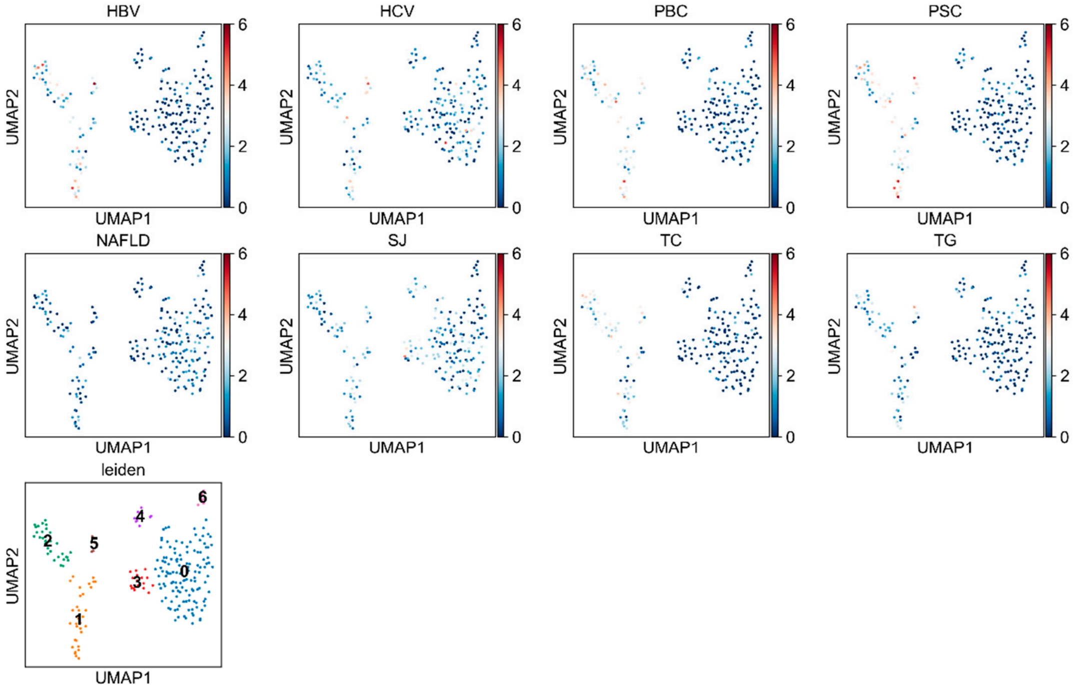

Disease score of LSECs

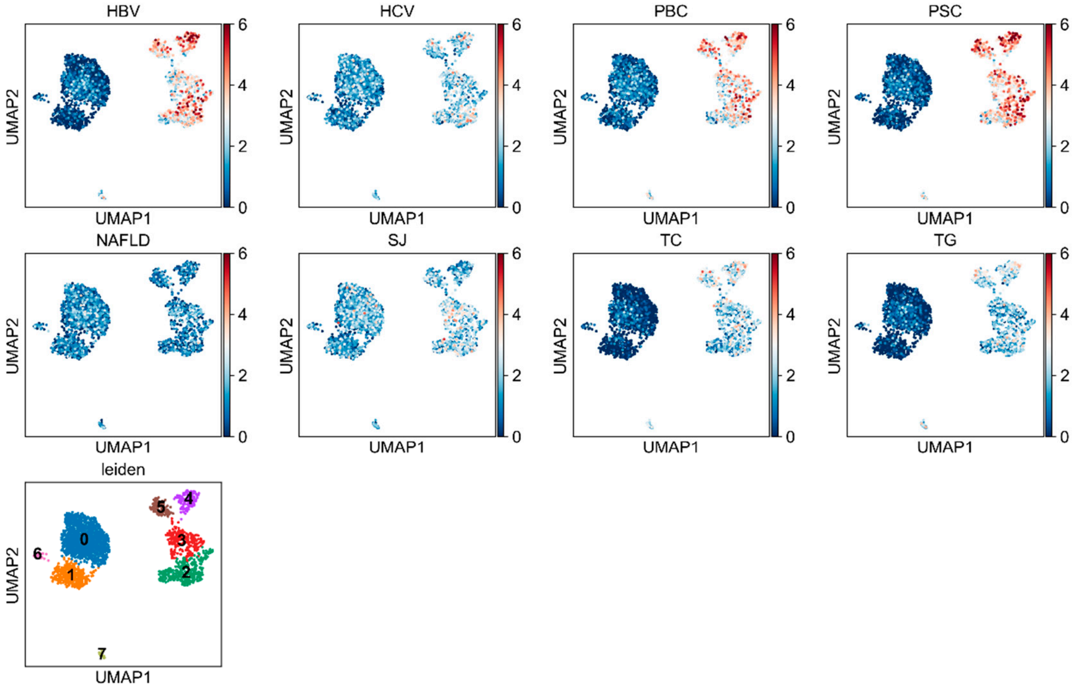

Disease score of Hepatocytes

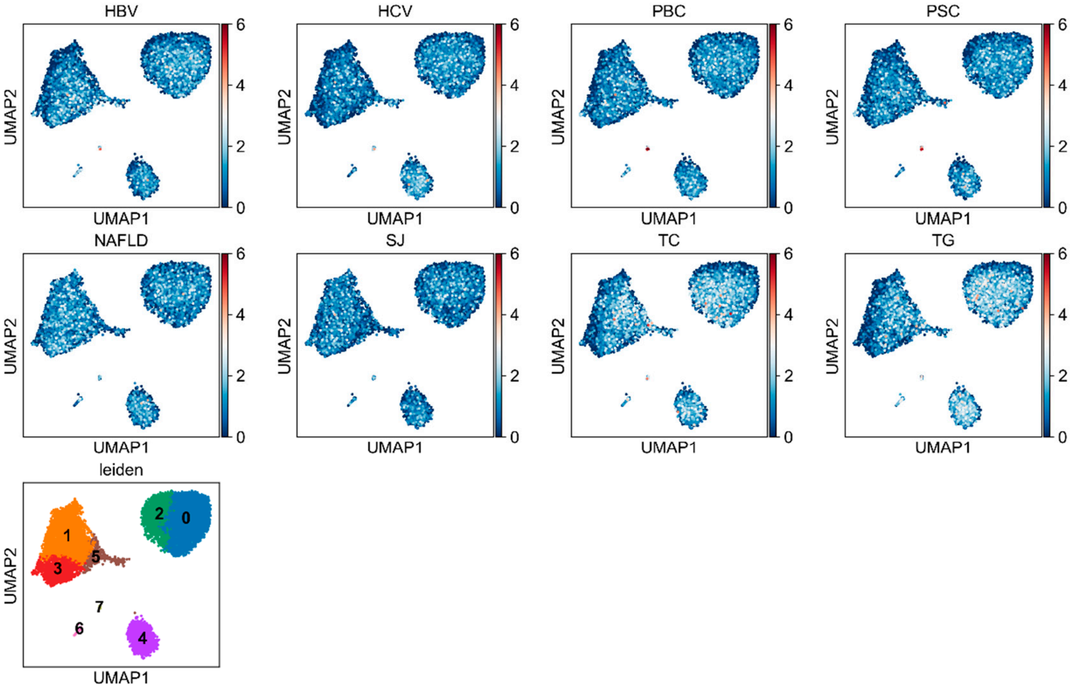

Disease score of Fibroblasts

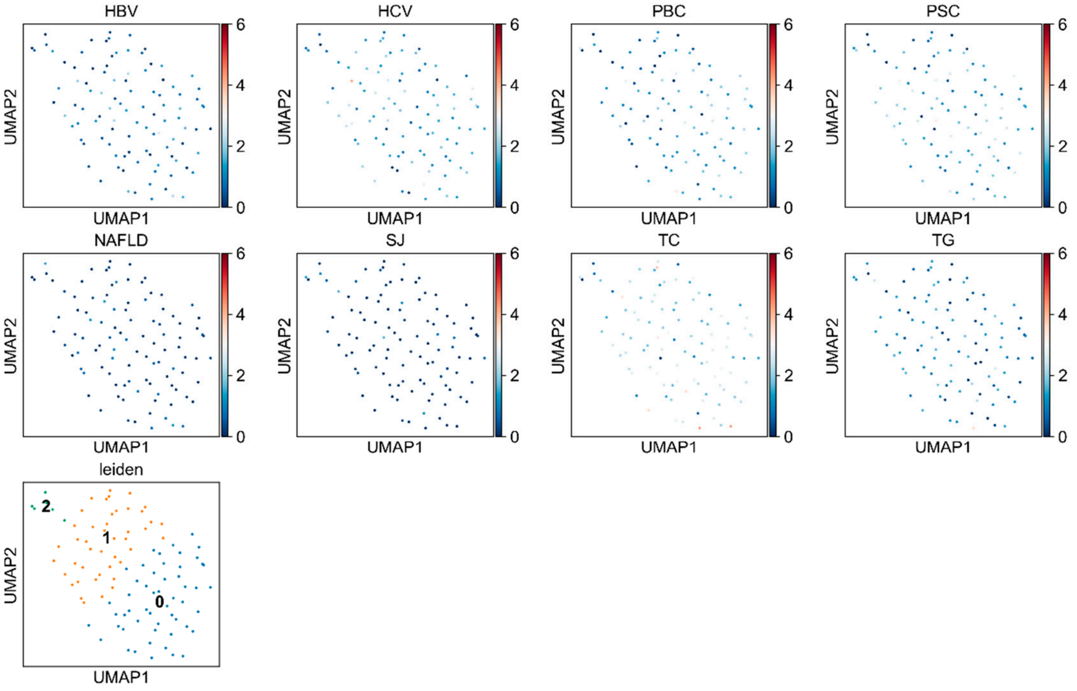

Disease score of Cholangiocytes

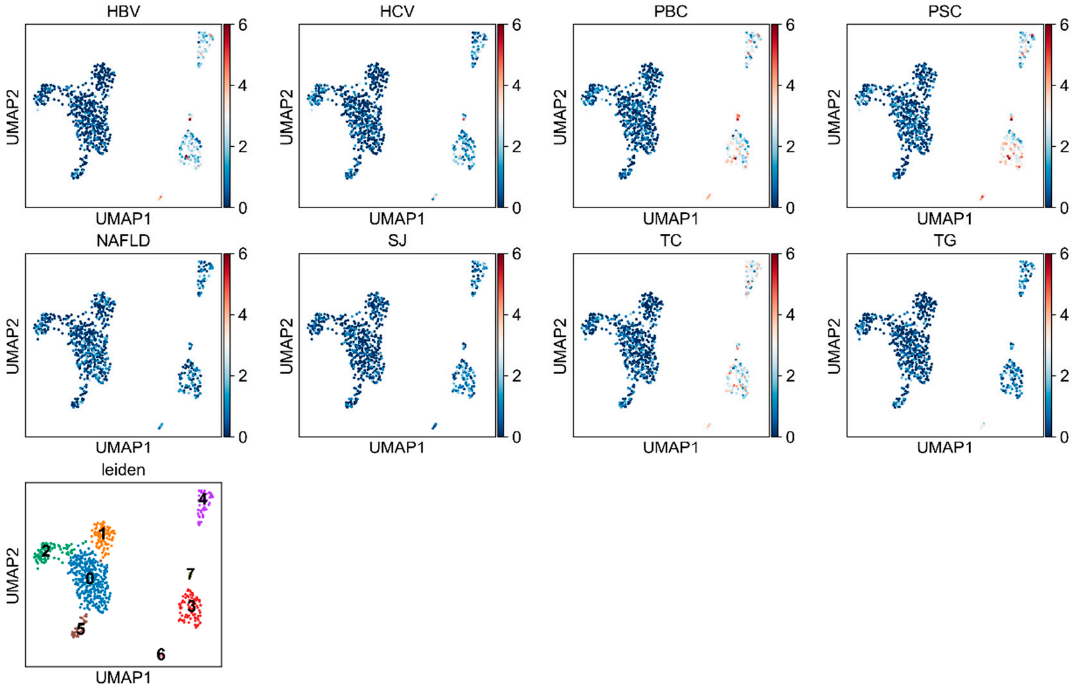

Disease score of Central Vein Endothelial cells

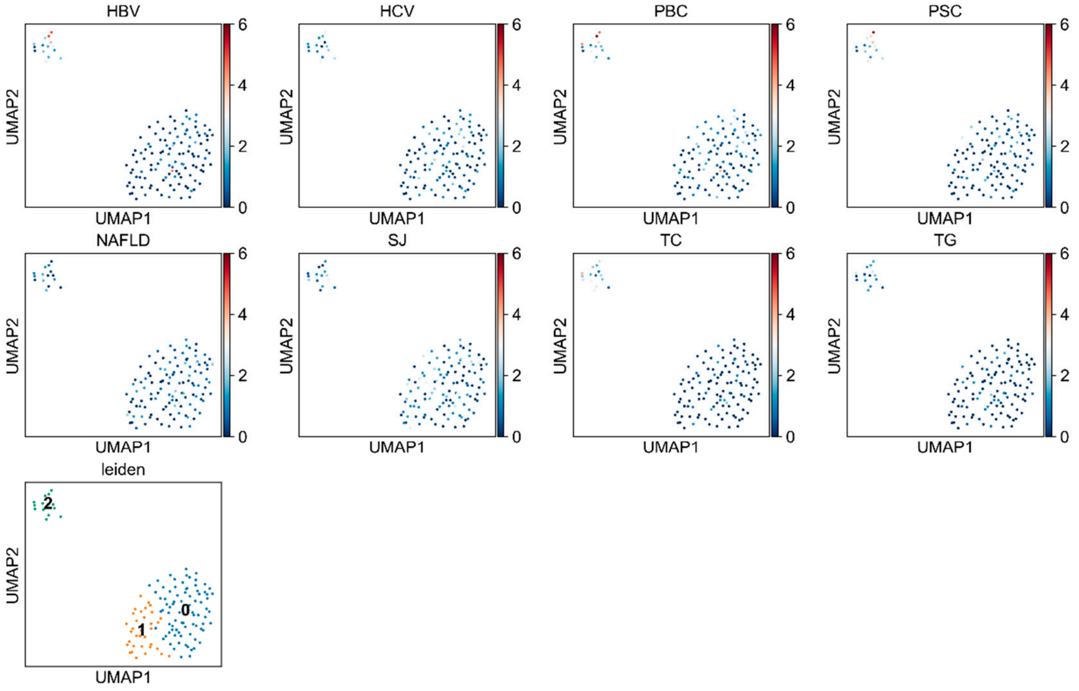

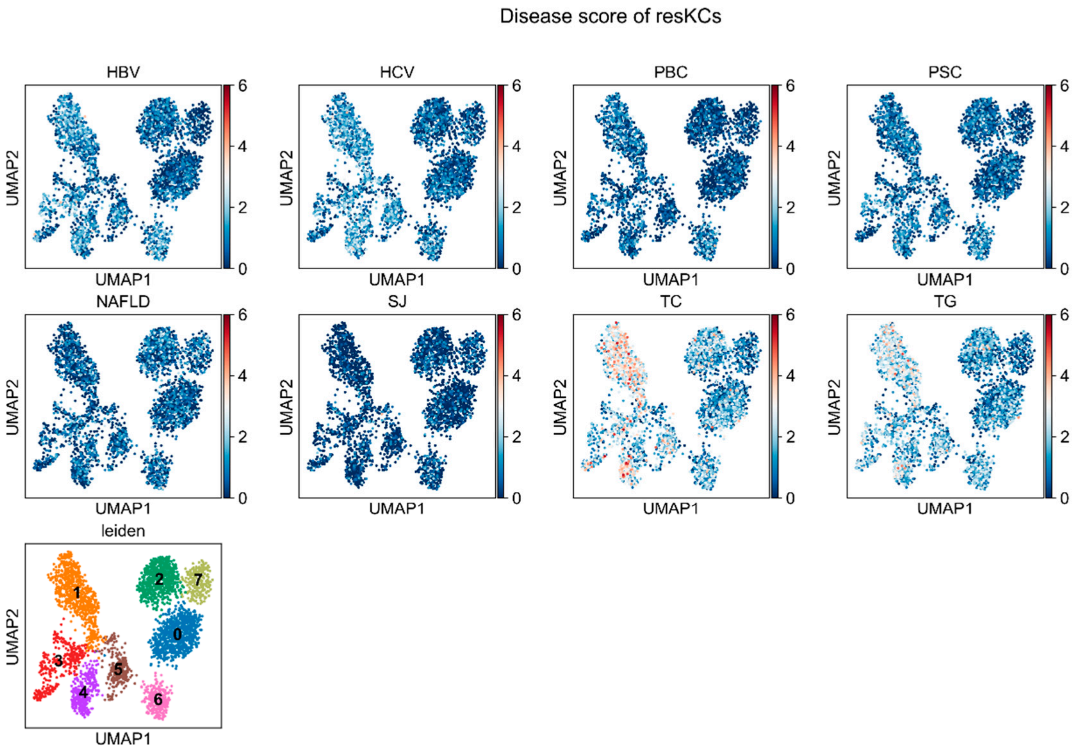

Figure S1. Associations between individual cells and disease within heterogeneous cell types. Each subpanel represents a specific cell type. "Leiden" indicates the clustering results obtained using the Leiden algorithm.
